# PReS-FINAL-2311: Rituximab in paediatric ANCA-associated vasculitis

**DOI:** 10.1186/1546-0096-11-S2-P301

**Published:** 2013-12-05

**Authors:** G Mestrallet, A Faye, P Quartier, B Ranchin, P Cochat, A Belot, SOFREMIP

**Affiliations:** 1 Pediatrics, CH Villefranche Hospital, Villefranche sur Saone, France; 2 Pediatric Rheumatology & Infectious Diseases Department, Hopital Robert Debré, Assistance Publique Hopitaux de Paris, Paris, France; 3 Pediatric Rheumatology department, Hopital Necker, Assistance Publique Hopitaux de Paris, Paris, France; 4 Pediatric Nephrology, Rheumatology & Dermatology Department, HOPITAL FEMME MERE ENFANT, HOSPICES CIVILS DE LYON, Lyon, France

## Introduction

*R*ituximab is an anti-CD20 monoclonal antibody that has proven to be effective in the treatment of ANCA-associated vasculitis (AAV) in adults. Standard treatments rely on corticosteroids and cyclophosphamide. Only isolated case reports of pediatric AAV treated by rituximab have been reported so far.

## Objectives

Our objective was to collect clinical data and outcomes of children with AAV treated with rituximab in a multicenter retrospective study.

## Methods

We conducted a retrospective study within the French paediatric rheumatology society (SOFREMIP) in 2011-2012.

## Results

We identified 6 children with AAV treated with rituximab (microscopic polyangeitis, n = 2, granulomatosis with polyangeitis, n = 2, unspecified vasculitis, n = 2). The age at onset ranged from 4 to 16 yrs. Treatment with rituximab consisted in 4 infusions of 375 mg/m^2^; one patient received only 3 infusions. Mean duration of follow-up was 2.65 yrs. All patients achieved clinical remission after 12 months. One patient presented several relapses associated with B cell increase and was dependent on rituximab infusions. No severe adverse event had been reported.

## Conclusion

From this multicenter retrospective series, short-term safety and efficicacy of rituximab in pediatric AAV is promising so that B cell depleting agents may represent an alternative to conventional treatment with cyclophosphamide but larger prospective studies are mandatory.

## Disclosure of interest

None declared.

